# Mechanisms of Action of Phytoestrogens and Their Role in Familial Adenomatous Polyposis

**DOI:** 10.3390/pharmaceutics16050640

**Published:** 2024-05-10

**Authors:** Irene Falsetti, Gaia Palmini, Teresa Iantomasi, Maria Luisa Brandi, Francesco Tonelli

**Affiliations:** 1 Department of Experimental and Clinical Biomedical Sciences, University of Florence, 50139 Florence, Italy; irene.falsetti@unifi.it (I.F.); teresa.iantomasi@unifi.it (T.I.); 2 Fondazione Italiana Ricerca sulle Malattie dell’Osso (FIRMO Onlus), 50129 Florence, Italy; gaia@fondazionefirmo.com (G.P.); marialuisa.brandi@unifi.it (M.L.B.)

**Keywords:** familial adenomatous polyposis, colorectal cancer, phytoestrogens, estrogen receptor β

## Abstract

Familial adenomatous polyposis (FAP) is a rare disease characterized by the development of adenomatous polyps in the colon and rectum already in adolescence. If left untreated, patients develop colorectal cancer (CRC) with a 100% probability. To date, the gold standard of FAP management is surgery, which is associated with morbidity and mortality. A chemopreventive agent capable of delaying, preventing and reversing the development of CRC has been sought. Several classes of drugs have been used but to date no chemopreventive drug has been found for the management of this disease. In recent years, the importance of estrogen receptors in FAP and CRC, particularly the β subtype, has emerged. Indeed, the expression of the latter is strongly reduced in adenomatous polyps and CRC and is inversely correlated with the aggressiveness of the disease. Since phytoestrogens have a high affinity for this receptor, they have been suggested for use as chemopreventive agents in FAP and CRC. A combination of phytoestrogens and insoluble fibres has proved particularly effective. In this review, the various mechanisms of action of phytoestrogens were analyzed and the effectiveness of using phytoestrogens as an effective chemopreventive strategy was discussed.

## 1. Introduction

Since the 1970s particular attention has been paid to nutrition and it has been realized that there is a relationship between diet and a lower risk of developing various types of cancer, including colorectal cancer (CRC). As a result of efforts in this field, it was claimed in the late 1990s that 30/40% of all cancer cases can be prevented by an adequate diet and regular physical activity, which allow one to maintain an optimal body weight [1].

Much attention has been paid to phytoestrogens, which belong to the large family of polyphenols. Phytoestrogens are defined as those polyphenol molecules of plant origin with a chemical structure similar to that of the main female sex hormone, 17-β-estradiol, and due to this structural similarity are able to bind to its estrogen receptors (ERs). They are secondary metabolites of plants, where they are produced in order to protect plants against pathogen attack, ultraviolet radiation and stress-related responses [2].

Phytoestrogens are cardioprotective, cholesterol-lowering, chemopreventive in Familial Adenomatous Polyposis (FAP), CRC, breast and prostate cancer; they can be used in the management of osteoporosis, atherosclerosis, stroke, diabetes, neurodegeneration and post-menopause [3].

Due to their chemopreventive properties in FAP and CRC, the aim of this review is to provide a general overview of phytoestrogens with a specific focus on FAP and their role in it.

## 2. Phytoestrogens

### 2.1. Classification and Sources of Phytoestrogens

Based on their chemical structure, phytoestrogens are classified into three main classes: isoflavones, coumestans and lignans [4].

Isoflavones are the most studied phytoestrogens. The main representatives of this class are genistein, biochanin A, daidzein, glycitein and formononetin. They can be found in fruit (apples, apricots, plums, melon, cherries), wheat, onions and blackcurrants, but the main dietary sources are soya foods [5]. Geographically, a high daily consumption of the latter naturally occurs in Asian countries, where it has been observed that 60% of the population (compared to 30% in Western countries) can metabolize daidzein into equol thanks to their intestinal microbiota. The latter has a higher affinity for ERs than daidzein [6].

Although they are not very common in the human diet, coumestanes are characterized by high estrogenic activity. In fact, coumestrol, one of the main phytoestrogens in this class, binds to ERs with an affinity similar to that of the natural ligand, 30 to 100 times higher than that of other phytoestrogens. Coumestans are found in alfalfa sprouts, beans and peas [5].

Lignans are widely distributed in the plant world. They are mainly found in the oilseeds flax, sesame and soya, whole cereals (wheat, rye, barley and oats), legumes, fruit and vegetables, wine, tea and coffee [7]. Lariciresinol, medioresinol, secoisolariciresinol, matairesinol, pinoresinol and syringaresinol belong to this class. Lignans are metabolized by bacteria in the intestinal flora, which produce enterolignans, such as enterodiol and enterolactone, to which the beneficial properties of lignans are attributed [8].

### 2.2. Mechanisms of Action of Phytoestrogens

Phytoestrogens are flat in shape, have at least one benzene ring and two or three hydroxyl groups placed at a similar distance to that existing in 17-β-estradiol between C3 and C17. This makes phytoestrogens related to 17-β-estradiol by a structural similarity and similar molecular weight. For these reasons, phytoestrogens are able to bind to ERs [9].

ERs are nuclear-type receptors that, following ligand binding, dimerize and translocate into the nucleus, where, by binding to promoter regions of DNA, they regulate transcription of target genes. They interact with numerous transcription factors, including nuclear factor kB (NF-kB), Sp1 and activator protein 1 (AP-1) [10].

There are two subtypes of ER, called ER-α and ER-β, described in 1985 and 1996 respectively, encoded by two different genes located on two different chromosomes. ER-α and ER-β are expressed in many tissues, which explains how 17-β-estradiol is involved in numerous processes in both physiological and pathological conditions in men and women (such as cancer, neuronal development, cardiovascular protection, bone density) [11,12]. However, ER-α and ER-β are characterized by different expression in these tissues. In particular, ER-α expression is higher in the uterus, breast, bone, cardiovascular and central nervous system, while ER-β expression is higher in the urogenital tract and intestine.

Although ER-α and ER-β share a high degree of homology, they promote signalling pathways with different cellular effects following 17-β-estradiol binding [13]. ER-α activates the extracellular signal-related kinases (ERKs)/mitogen-activated protein kinases (MAPK), phosphatidylinositol-3-kinase/protein kinase B (Akt) signalling pathways, which promote cell growth, whereas ER-β triggers apoptosis in tumour cells via caspase 3 activation, phosphorylation of p38/MAPK and PARP cleavage, blocks the cell cycle by increasing the expression of the oncosopressors p21 and p27 and decreasing that of the protooncogenes c-myc and cyclins [14,15]. 

The biological effect depends, therefore, on which receptor subtype is involved, but in general it can be observed that the proliferative effects promoted by ER-α activation are antagonized by the pro-apoptotic effects promoted by ER-β activation.

In addition to these genomic effects, phytoestrogens are able to exert effects independent of ERs binding, which are called non-genomic effects. These include inhibition of tyrosine kinase and DNA topoisomerase, angiogenesis, antioxidant effects, risk reduction of various cancers, activation of insulin-like growth factor-I (IGF-1) and serotonergic receptors, interacting with cAMP/protein kinase A, cGMP/nitric oxide (NO), phosphatidylinositol-3 kinase/Akt and MAP (ERK1,2, p38) kinases and transcription factors (including NF-kB), influencing RNA expression, DNA methylation, cell cycle and apoptosis regulators and histone modifications [16,17]. 

Phytoestrogens have antioxidant properties as they promote the transcription of genes coding for antioxidant enzymes (catalase (CAT), superoxide dismutase (SOD) and glutathione peroxidase) [18]. Secoisolariciresinol diglucoside resulted in up-regulation of the expression of SOD and CAT in the liver and kidneys of damaged mice, as well as an increase in glutathione levels. Another mechanism by which secoisolariciresinol diglucoside exerts antioxidant effects has been shown to be the activation of nuclear factor erythroid 2-related factor 2 [19].

Phytoestrogens possess anti-inflammatory properties. Lignans and their metabolites exert these properties through the inhibition of the transcription factors Janus kinase-signal transduction and activator of transcription, NF-kB, AP-1, which lead to an up-regulation of pro-inflammatory factors, including Tumour Necrosis Factor-α, interleukins 6 and 1β, COX-2 and inducible NO synthase [19]. 

Phytoestrogens, particularly those derived from sesame and linseed, act as metastasis inhibitors. The mechanism of action seems to be related to the regulation of autophagy, in particular, dietary lignans inhibit UNC-51-like autophagy activating kinase 1/2 [20]. Sesamin, a phytoestrogen belonging to the lignan family, has been observed to act as a metastasis inhibitor in CRC by blocking angiogenesis [21].

One of the best-known phytoestrogens is genistein, which has many properties. It has antioxidant properties, is an inhibitor of angiogenesis and metastasis, NF-kB and Akt, influences the expression of genes involved in the cell cycle and apoptosis [19]. Furthermore, the anti-proliferative and pro-apoptotic effects of genistein in colon cancer cells, in addition to its interaction with ER-β, can be attributed to its ability to activate caspase 3, FOXO3, and to negatively influence the activity of tyrosine kinase, epidermal growth factor and IGF-1 receptors [4]. 

Genistein is a DNA methyltransferase inhibitor. It has been shown to inhibit DNA methylation at the promoter level of WNT genes, which plays an important role in carcinogenesis. In colon cancer, loss of WNT expression occurs due to hypermethylation of the promoter or hyperactivation of the signalling pathway. It can be concluded that genistein may have a chemopreventive effect in the development of CRC due to its action on the WNT pathway [6].

Genistein binds to both ERs but has a higher affinity for the β subtype. In fact, Rietjens et al. performed a literature review to study binding affinity as IC_50_ values from competitive binding assays of 17-β-estradiol and phytoestrogens to ERs and found that phytoestrogens have higher IC_50_ values for both ER-α and ER-β than 17-β-estradiol, indicating that they are less potent than 17-β-estradiol itself [22]. Furthermore, the phytoestrogens analyzed (with very few exceptions) have higher IC_50_ values for ER-α than for ER-β and have a preference for ER-β-mediated gene expression [22]. This results in a higher binding affinity of the phytoestrogens for ER-β. 

These data collectively demonstrate that phytoestrogens have a higher affinity for ER-β, and since ER-β has been shown to play an important role in the development of adenomatous polyps and in the early stages of CRC carcinogenesis, the efficacy of selective agonists for this receptor subtype in the chemoprevention of FAP and CRC was investigated [23,24].

In fact, ER-β agonists have received a great deal of attention because their use does not lead to an increased risk of cardiovascular or cerebrovascular complications or even breast or endometrial cancer, which are more likely to occur during the use of oral contraceptives [25]. 

## 3. Familial Adenomatous Polyposis

FAP is an autosomal dominant inherited disease due to an inactivating mutation in the oncosuppressor gene *Adenomatous Polyposis Coli* (APC) [26]. It is characterized, already in the first or second decade of life, by the appearance of numerous precancerous polyps of different sizes in the mucosa of the colon and rectum, which tend to increase in number and size over time. 

In addition, FAP is accompanied by numerous extra-colonic manifestations, such as polyps and tumours of the upper gastrointestinal (GI) tract, desmoid tumours, congenital hypertrophy of the retinal pigment epithelium, hepatoblastoma, thyroid tumours, osteomas of the maxillary bones, skull and long bones, supernumerary teeth, sebaceous cysts and adenomas of the adrenal gland. Some of these may arise even before colon manifestations and are still a cause of death for these patients today [27]. 

The duodenum, immediately following the colon and rectum, is the other anatomical district most affected by polyposis [28]. In fact, the risk of developing duodenal polyps is almost 100% in FAP patients. The most affected portions of the duodenum are the second and third, but the area with the greatest degree of malignancy is the periampullary. 

The duodenal ampullary adenocarcinoma affects approximately 4–12% of FAP patients as early as adolescence and still represents a major cause of death [29]. The risk of developing duodenal cancer in FAP patients is 100–300 times higher than in the general population. Fortunately, however, in FAP patients the pre-malignant state is often detected thanks to periodic endoscopic surveillance of the upper GI tract [30]. 

However, when duodenal adenomatosis becomes severe and no longer treatable by endoscopic treatment, surgical procedures may be used. Ampullectomy and duodenotomy with local excision of polyps guarantee few complications, thus eliminating the risk of developing cancer. This type of technique can be used in the presence of severe adenomatosis in young patients. On the other hand, in case of rugged polyposis, more extensive surgical techniques are needed, such as pylorus-preserving pancreatoduodenectomy and pancreas-sparing duodenotomy, which are however associated, with recurrence of polyps in the small intestine, lead to significant morbidity and in some cases death [31]. 

FAP patients also develop fundic gland and jejunal polyps. The former is the most common type of gastric polyp. They arise in the body and fundus of the stomach in FAP patients and are usually benign [32]; the latter have a low malignant potential and transformation to adenocarcinoma, although rare, has been found in older patients [33]. 

Patients with FAP also appear to have an increased risk of developing Barrett’s esophagus and esophageal adenocarcinoma, due to inherited mutations in the gene responsible for FAP [34]. However, they are rarer than other extracolonial manifestations [35]. If FAP does not come to be treated, the risk of developing CRC increases with age and is almost 100% around the age of 35–45 years; it is thus responsible for 1% of all CRC cases. The transformation from polyp to cancer occurs over a period of time in a multistep process that may vary from 10 to 15 years depending on the size of the polyp, its degree of dysplasia and its villous histology [36]. This multistep process is the result of mutations in several genes that are involved in the regulation of important processes such as growth, differentiation and programmed epithelial cell death. 

### 3.1. Genetics

Mutations in the *APC* gene, located on the long arm of chromosome 5 in the q21 band (5q21), are responsible for the onset of FAP. 

*APC* consists of 15 exons (of which the fifteenth constitutes the largest coding region), forming a coding sequence of 8972 bp [37]. *APC* is expressed in many tissues, both foetal and adult, including colorectal epithelium. Its codes for a protein consisting of 2843 amino acids and weighing 312 kDa, whose structure contains both functional and structural sites through which it interacts with other proteins [38].

*APC* is an oncosuppressor, which takes part in the degradation of β-catenin, an intracellular protein, acting as a downstream transcriptional activator in the WNT signalling pathway [39]. Furthermore, *APC* regulates cell migration and adhesion, the cell cycle, differentiation, apoptosis, cytoskeleton reorganization and chromosome stability during mitosis [37,40,41].

The mechanism of inheritance is commonly autosomal dominant with a very high penetrance, almost 100%. A lower percentage (30%) of FAP cases are due to de novo mutations. In these cases, it may be more difficult to make a diagnosis of FAP as there is no family history [26]. 

To date, more than 2500 germline mutations have been identified in the *APC* gene of patients with FAP [42]. The most common mutations are nonsense or frameshift, which result in the insertion of stop codons, leading to a truncated protein. They can be found throughout the coding region but cluster mainly at the mutation cluster region, i.e., at the 5′ end of exon 15 [32]. 

It has been observed that there is a genotype-phenotype correlation, i.e., the clinical manifestations of the disease depend on the location of the mutation. Mutations in codon 1309 have been correlated with an increased aggressiveness of the disease, resulting in the development of a greater number of polyps (up to 5000) and the onset of both colonic and CRC events occurs at a younger age than the average [43,44,45]. 

### 3.2. Pharmacological Treatment

Surgical resection of the colon represents the safest standard of care in FAP patients. It is difficult to identify the right time to resort to surgery, because it should affect patients’ quality of life as little as possible but at the same time should prevent the development of CRC [46]. The guidelines state that surgery should be performed immediately for FAP patients with a confirmed or pending diagnosis of CRC or with symptoms and recommend surgery if there is an increase in the number of polyps or when multiple adenomas greater than 6 mm are present [47]. 

There are two main prophylactic surgical techniques, colectomy with ileorectal anastomosis and proctocolectomy with ileal pouch-anal anastomosis (IPAA) [48]. The most used surgical technique is IPAA because, although it is associated with more post-operative obstacles and a longer convalescence period, it carries a lower risk of developing rectal cancer [49]. 

Although both techniques have been improved in recent years, they entail major life changes and are still associated with increased morbidity rates on urological, bowel, sexual and fertility function [50,51,52]. 

The need to undergo invasive examinations, the need for surgery, with the consequent periodic surveillance examinations, the increased risk of post-operative complications and the continued progression of the disease have prompted research to search for an effective chemopreventive treatment for the chronic management of FAP [53].

Chemoprevention consists of the long-term use of a wide variety of substances, both of chemical and natural origin, with the aim of delaying, preventing and, if possible, reversing the multistep process that leads to the development of cancer. Thus, one can apply this strategy before the development of cancer as prevention or at the time when the first changes in the cells begin to occur to slow down the multistep process but also to delay the recurrence of a new tumor [54]. The ideal chemopreventive agent must be non-toxic (because it is administered over long periods), effective even at low doses, inexpensive, easily administered and available. 

Chemoprevention in FAP aims to delay prophylactic colectomy, reduce the burden of polyps in the colorectum to slow down the development of CRC and extra-colonic manifestations, such as cancer in the upper GI tract, particularly at the duodenal level [53,54].

Over the years, many efforts have been made to find the ideal chemopreventive drug for the treatment of FAP. One of the most studied classes is the non-steroidal anti-inflammatory drugs (NSAIDs), which inhibit the enzyme cyclooxygenase (COX), of which two isoforms are known in humans (constitutive COX-1 and inducible COX-2). The rationale for their use in FAP stems from the demonstration that COX-2 expression is increased by 40/50% in adenomas and 80% in CRC, while it is absent or expressed at low levels in normal colonic mucosa [55]. It has been observed that COX-2 over-expression is dependent on distal site, increased adenomatous polyp burden, high degree of dysplasia and polyp size [56].

For these reasons, the pharmacological properties of NSAIDs and selective COX-2 inhibitors were evaluated in animal models of FAP and subsequently in clinical trials in patients with FAP. The results of these trials were so encouraging that the Food and Drug Administration approved celecoxib and rofecoxib, two selective COX-2 inhibitors, as adjuvants in FAP patients.

However, despite initial enthusiasm, significant cardiovascular toxicity has emerged during their clinical use. A dose-dependent increase in deaths from cardiovascular problems, such as heart failure, stroke and non-fatal myocardial infarction, has been reported in patients treated with the COX-2 selective drugs [57,58,59]. 

This cardiovascular toxicity has proved so limiting to therapeutic use that some pharmaceutical companies have voluntarily decided to withdraw their products from the market. As of 2011 celecoxib is no longer considered the chemopreventive drug of first choice for FAP and in fact the indication for therapeutic use in FAP has been removed from its label [60]. Thus, overall this class of drugs is not the ideal chemopreventive class for the treatment of FAP due to significant toxicity. 

Other substances with different mechanisms of action have also been tested (including D,L-α-difluoromethylornithine and ω-3 polyunsaturated fatty acids), but none of them can be considered the ideal chemopreventive drug for FAP [61,62,63,64,65,66,67].

To date, no drug has been identified for the pharmacological management of FAP. In recent years, however, the role of ERs, in particular the β subtype, in FAP and CRC has been thoroughly delineated. 

In fact, ER-β is expressed in healthy colonic mucosa, where it maintains homeostasis, controls epithelial architecture and GI physiology, participates in immune and anti-inflammatory responses. Its expression, however, decreases dramatically in adenomatous polyps and CRC and this is associated with increased intestinal permeability [15,23]. Indeed, ER-β-mediated chemopreventive action of estrogen has been shown to be active in the initiation and promotion phase of CRC [13].

It has been shown that in the transition from normal mucosa to neoplasm there is a progressive decrease in ER-β levels and a simultaneous progressive increase in ER-α levels. The ratio of anti-carcinogenic ER-β to pro-carcinogenic ER-α is decreased [68]. 

Moreover, ER-β expression is inversely correlated with proliferative activity and disease aggressiveness [23,24]. 

## 4. Phytoestrogens in FAP

Numerous studies have been conducted in order to evaluate the efficacy of phytoestrogens in FAP and CRC, as more natural substances have been used in recent years rather than synthetic drugs [69]. 

Javid et al. [70] tested the effects of 17-β-estradiol, genistein and coumestrol, which represent an alternative to hormone replacement therapy, in ovariectomized Min mice. Genistein, which was administered at a high concentration in the diet, did not demonstrate chemopreventive properties because it resulted in a non-significant reduction in the number of intestinal tumours. The authors hypothesized that this lack of effect was due to the numerous enzymatic reactions that genistein undergoes in the GI. These, in fact, lead to the synthesis of heterocyclic phenols with an estrogen-like structure, but their concentrations can be very different between mice on the same diet due to the diversity of GI flora and reabsorption in the enterohepatic circulation. Also in another in vivo study in ovariectomized APC Min mice, genistein combined with a high-fat diet did not result in significant reductions in the number or size of intestinal tumours [71]. Coumestrol, on the other hand, resulted in a reduction in the number of tumours compared to controls. The effect of orally administered coumestrol demonstrated preventive properties in tumourigenesis in these mice comparable to those of 17-β-estradiol administered subcutaneously. In addition, both coumestrol and 17-β-estradiol resulted in an enhanced association of E-cadherin and β-catenin proteins and increased expression of ER-β in enterocytes of ovariectomized Min mice [56].

Also in ovariectomized female mice, the effect of silymarin was tested, which is a mixture of four flavonolignans (silybinin, isosilybinin, silydianin and silycristin) and the isoflavonoid taxifolin and is extracted from Silybum marianum. Seidlová-Wuttke et al. demonstrated in animal model that silymarin components only bind to ER-β and can therefore be considered specific agonists for this receptor [72]. The chemopreventive properties of dietary silymarin were also demonstrated by Kohno et al., who administered it to male F344 rats in which carcinogenesis in the colon was chemically induced with azoxymethane [73]. In the short-term study, during and after exposure to carcinogens, administration of silymarin for 4 weeks resulted in a dose-dependent decrease in the frequency of aberrant crypt foci in the colon and an increase in the activity of detoxifying enzymes. In the long-term administration in the early phase of carcinogenesis, the addition of silymarin to the diet decreased both the incidence and multiplicity of adenocarcinoma in the colon. 

Barone et al. added silymarin and lignin (a dietary fibre) to the high-fat, low-fibre diet of Apc Min mice and observed that this combination was able not only to reduce the number and volume of colon polyps and improve the degree of dysplasia of polyps, but also to up-regulate the expression of ER-β without altering that of ER-α [74].

Zhang et al. studied the effects of genistein in rats with colon cancer chemically induced by azoxymethane in both the pre-cancerogenesis and post-cancerogenesis periods, thus including both gestation and lactation [75]. Genistein reduced the number of aberrant crypts. In addition, genistein prevented the hyper-activation of the WNT signalling pathway, which was induced by azoxymethane, by restoring it to levels prior to azoxymethane treatment. β-catenin, c-Myc and Cyclin D1 were also strongly reduced by genistein. These data taken together indicate the protective role of genistein following prolonged administration and suggest that the mechanism by which genistein acts is through regulation of WNT/β-catenin signalling.

The down-regulation of WNT/β-catenin signalling by genistein has also been demonstrated in HT-29, a human adenocarcinoma cell line [76].

The aim of the work by Bises et al. was to evaluate in C57BL/6 mice the safety of long-term administration of soy (of which an important constituent is genistein) for two generations and how this would alter markers of apoptosis, proliferation and inflammation and highlight any differences between males and females [77]. No differences in results were found between the first and second generation, so the soybean was safe. Differences in the levels of the various markers were found between male and female mice. In males, the administration of soy did not change the expression levels of Bak (which is pro-apoptotic), whereas in females a decrease in Bak levels was recorded only in the proximal colon. Also in females there was an increase in COX-2 expression in the proximal colon. Another difference occurred in the expression levels of the two ERs. Indeed, in both males and females there was no difference in ER-β levels, but in females there was an increase in ER-α mRNA. Thus, in female mice, exposure to a component of soy, not yet identified, could be detrimental on the colonic mucosa due to the background of estrogen. 

A difference between male and female mice was also reported by Oikarinen et al., who studied the effects of flaxseed in Min mice [78]. Specifically, higher levels of enterolactone were found in the small intestine and cecum of male Min mice treated with flaxseed compared to female Min mice. These intestinal gender differences in enterolactone levels were not found in plasma. Surprisingly, there was no change in the number, distribution, size or incidence of adenomas between the treated and control group, only a decreasing trend in the number of adenomas in the flaxseed-treated group. 

van Kranen et al., on the other hand, demonstrated that flaxseed lignan precursors had no protective effect in Apc Min mice fed a diet high in animal fat and low in calcium [79]. 

Girardi et al. evaluated the preventive effect of a nutritional formulation enriched with silymarin, boswellic acid and curcumin in Apc Min/+ animals [80]. The number and size of polypoid lesions were smaller in the small intestine of mice on the enriched diet than in the control group. No difference in ER-α expression was observed between normal and polypoid tissue. ER-β expression was low in the polyps of both groups, but was higher in the healthy mucosa of the enriched diet group. The authors demonstrate the efficacy of a combined plant-based diet in preventing intestinal carcinogenesis. 

An interesting dietary supplement is a patented mixture of dietary phytoestrogens (silymarin and lignans) and insoluble fibres, which were previously tested individually in rodents and produced beneficial effects, such as reduction in the size and number of polyps, induction of apoptosis, inhibition of cell proliferation, inflammation and angiogenesis [81,82,83,84]. 

Principi et al. [85] tested this mixture of phytoestrogens and insoluble fibres. They conducted a randomized, double-blind, placebo-controlled study to evaluate the effect of this mixture on the levels of ER-β and other biological markers in the colon mucosa of 60 men and post-menopausal women who had previously undergone surveillance colonoscopy for sporadic colonic adenomas. The study was conducted in two phases. In the first phase, a significant increase in ER-β protein levels and a non-significant increase in ER-β mRNA levels was found in the group treated with this phytoestrogen mixture compared to the control group. No differences in ER-α protein and mRNA were found between the two groups. In fact, in the treated group, there was an increasing trend in the ratio of ER-β/ER-α, with increased rates of apoptosis (assessed by TUNEL and caspase 3) and epithelial proliferation. The second phase did not indicate that the use of the mixture would be helpful in preventing the recurrence of polyps, but this may also be due to the short period of administration (two months) and the small number of study participants. However, no adverse effects were reported in either group. The importance of ER-β as a biomarker of CRC risk thus emerges from this work. 

In the same year, another group tested the same mixture on FAP patients with IPAA and recurrent adenomas in the duodenal mucosa [86]. Eleven patients were enrolled and orally administered 5 mg of the phytoestrogen mixture twice a day for 90 days. This administration led to a 32% reduction in the number of polyps and a 51% reduction in their size. All patients completed the study, confirming the safety and tolerability of the treatment; only one patient experienced slight intestinal swelling but this did not lead to discontinuation of therapy. 

Bringiotti et al. [87] administered 2 sachets per day of the mixture of phytoestrogens and insoluble fibres to a 55-year-old man who had previously undergone right haemicolectomy for Lynch syndrome. Already after 3 months, a statistically significant reduction in the number and size of polyps was observed, which was further confirmed in the following 3 months of treatment. After 9 months, no changes were observed in the oesophagus and stomach. This study demonstrates the chemopreventive power of this mixture. 

The aim of a study by Calabrese et al. [88] was to evaluate whether the administration of 5 mg of the patented mixture of phytoestrogens and insoluble fibres twice daily for 3 months in 15 FAP patients with IPAA resulted in a decrease in the number of duodenal polyps and a change in gene expression. After 3 months, there was a reduction in both the number and size of duodenal polyps in all patients. Important changes were also recorded in the expression of CRC promoter and inhibitor genes. In fact, initially the expression of proliferating cell nuclear antigen (PCNA), mucin- (MUC) 1 and COX-2 (promoters) was up-regulated, while that of MUC-2 and ER-β (inhibitors) was down-regulated; after treatment, the expression of PCNA and COX-2 decreased significantly, that of ER-β increased to healthy mucosal values. The expression of MUC-2 and caveolin-1 also increased. This gene modulation, which occurred at the level of the lesions but not in the healthy mucosa, was further confirmed by studying the expression of miR-101, an oncosuppressor of CRC carcinogenesis. Following administration, miR-101 expression was found to be increased. Again, only one patient experienced slight intestinal swelling, but still completed the study. 

Seventeen patients diagnosed with FAP due to positivity to at least two selected criteria (presence of a germline mutation of the *APC* or *MYH* genes, presence of at least 100 polyps in the colon and family history of multiple adenomatous polyps in the colon) were enrolled in the study conducted by Tonelli et al. [89]. Ten patients were treated with 5 g of the phytoestrogen-fibre mixture twice a day for 12 months and seven patients for more than a year. After only 6 months of treatment, there was a significant reduction in the number of polyps; after 12 months the number of polyps decreased in 5 patients and remained stable in another 5, and further decreased in the 7 patients who continued treatment for more than 12 months. The expression of the two ERs was assessed after 6, 12, 18 and in some patients 24 months of treatment. At the level of the intestinal mucosa of the rectum and ileal pouch, the expression of ER-β increased significantly after several months of treatment. Interestingly, this up-regulation occurred in parallel with the decrease in both the number and size of polyps and the degree of dysplasia. The expression of ER-α did not change following treatment, confirming that its components selectively act on ER-β. Not only did patients experience no side effects, but removal of recurrent polyps was not necessary for more than two years for all but one study participant. 

Luceri et al. [90] evaluated the effect of the mixture of phytoestrogens and insoluble fibres on ER-β expression and spontaneous tumourigenesis in the colon of Pirc rats. These were treated from the first month of age and were sacrificed at the fourth month. In the colon of treated rats, the number of pre-neoplastic lesions and macroscopic tumours was lower than in the control group, while the number of apoptotic cells in the tumours and the expression of BOK (a pro-apoptotic gene) were higher in the treated group than in the control group. Also in this study, treatment with this mixture resulted in a significant increase in the expression of ER-β in colon tumours, while it did not affect the expression of ER-α. This mixture reduced tumorigenesis in the colon of Pirc rats, leading to an increase in apoptosis and ER-β expression.

Overall, these studies show that phytoestrogens and ER-β agonists in general can not only play a protective role in FAP, but also that they are very selective for this receptor subtype and therefore their use does not lead to the occurrence of any relevant side effects. The mixture of phytoestrogens and insoluble fibres showed interesting carcinogenesis-preventing and ER-β up-regulating effects in both animal models and FAP patients, good tolerability and minimal undesirable side effects. For these reasons, it is now an effective chemopreventive supplement available for therapeutic application. 

Table 1 summarizes the effects of the phytoestrogens and mixture of phytoestrogens and insoluble fibres in FAP.

## 5. Conclusions

Over the years, many efforts have been made to find optimal treatments for FAP, a rare disease that predisposes to CRC if left untreated. Several acting drugs have also been tested, but none of them reflect the characteristics of an ideal chemopreventive drug. In recent years, it has been observed that the expression of ER-β decreases dramatically in the colonic mucosa during FAP or CRC and therefore the effects of phytoestrogens have been studied. The results of studies in models of FAP and CRC are encouraging. Certainly, phytoestrogens are more tolerable following long-term administration, with no (or at least minimal) side effects, and the therapeutic effects obtained from their use are often comparable to those demonstrated by COX-2 selective drugs. An interesting product that has been established as a chemopreventive agent for FAP and CRC is a patented combination of phytoestrogens and insoluble fibres. A few recent studies have shown that this mixture is not only able to reduce carcinogenesis in both rats and humans, but also to up-regulate ER-β expression and induce apoptosis, as well as being safe and well-tolerated. However, to date, few in vitro studies have been conducted to understand in detail its functionality at the cellular and molecular level of neoplastic tissues, i.e., what is its mechanism of action directly on the polyp. Moreover, the clinical studies that will be necessary to effectively define this combination as a valid chemopreventive drug in the treatment of FAP, and consequently also of CRC, are still lacking.

## Figures and Tables

**Table 1:** Summary of the effects of phytoestrogens and mixture of phytoestrogens and insoluble fibres in FAP. ER-β: estrogen receptor β; ER-α: estrogen receptor α; COX-2: cyclooxygenase; PCNA: proliferating cell nuclear antigen; MUC-2: mucin-2; /: no data available.

Species	Phytoestrogens or Compounds	Effects	Expression of ERs	Reference
Mice	17-β-estradiol, genistein, coumestrol	Coumestrol reduced the number of tumours	Coumestrol and 17-β-estradiol increased ERβ experession	[70]
Rats	Silymarin	Decrease in the frequency of aberrant crypt foci and increase in the activity of detoxifying enzymes;	/	[73]
		Decrease the incidence and multiplicity of adenocarcinoma in the colon		
Mice	Silymarin and lignin	Decrease in number and volume of polyps;	Increased expression of ER-β;	[74]
		Improvement in the degree of polyps dysplasia	No change in expression of ER-α	
Rats	Genistein	Decrease in number of aberrant crypts;	/	[75]
		Prevention of hyper-activation of the WNT signalling pathway;		
		Decrease in the levels of β-catenin, c-My and Cyclin D1		
HT-29	Genistein	Down-regulation of WNT/β-catenin signalling	/	[76]
Mice	Soy	Decrease in Bak levels in females;	No change in expression of ER-β;	[77]
		Increased expression in COX-2 in female	Increased expression of ER-α in females	
Mice	Flaxseed	Decreasing trend in the number of adenomas	/	[78]
Mice	Silymarin, boswellic acid and curcumin	Decrease in number and size of polypoid lesions	No change in expression of ER-α;	[80]
			Increased expression of ER-β	
Patients	Silymarin, lignans and insoluble fibres	Increased apoptosis and epithelial proliferation	Increased levels of ER-β protein;	[85]
			No change in protein levels and expression of ER-α	
Patients	Silymarin, lignans and insoluble fibres	Decrease in number and size of polyps	/	[86]
Patients	Silymarin, lignans and insoluble fibres	Decrease in number and size of polyps	/	[87]
Patients	Silymarin, lignans and insoluble fibres	Decrease in number and size of polyps;	Increased expression of ER-β	[88]
		Decreased expression of COX-2 and PCNA;		
		Increased expression of MUC-2 and caveolin-1		
Patients	Silymarin, lignans and insoluble fibres	Decrease in number of polyps	Increased expression of ER-β;	[89]
			No change in expression of ER-α	
Rats	Silymarin, lignans and insoluble fibres	Decrease in number of pre-neoplastic lesions and macroscopic tumours;	Increased expression of ER-β;	[90]
		Increased expression of BOK	No change in expression of ER-α	

## Data Availability

Not applicable.
